# Rapid, specific, and sensitive detection of the *ureR_1* gene in *Klebsiella pneumoniae* by loop-mediated isothermal amplification method

**DOI:** 10.1590/1414-431X20198186

**Published:** 2019-03-25

**Authors:** Chao Li, Gongyu Fu, Yaoqiang Shi, A-Mei Zhang, Xueshan Xia, Yue Fang, Xiaoqin Mao, Jie Jiang, Yuzhu Song, Guangying Yang

**Affiliations:** 1 Faculty of Life Science and Technology, Kunming University of Science and Technology, Kunming, Yunnan, China; 2 Molecular Medicine Center of Yunnan Province, Kunming, Yunnan, China; 3 Department of Clinical Laboratory, First People's Hospital of Yunnan Province, Kunming, Yunnan, China; 4 Yunnan SciSpark Biotechnology Co. Ltd., Kunming, Yunnan, China

**Keywords:** Loop-mediated isothermal amplification, Polymerase chain reaction, Klebsiella pneumoniae, Novel specific gene, Specific, Sensitive method

## Abstract

*Klebsiella pneumoniae* is one of the main pathogenic bacteria that causes nosocomial infections, such as pneumonia, urinary tract infection, and sepsis. Therefore, the rapid and accurate detection of *K. pneumoniae* is important for the timely treatment of infectious patients. This study aimed to establish a loop-mediated isothermal amplification (LAMP) method for the rapid and sensitive detection of *K. pneumoniae*-specific gene *ureR_1* (Gene ID: 11847803). The *ureR_1* gene was obtained through local and online BLAST, and the specific primers were designed for its detection. Positive reactions were observed on all 140 *K. pneumoniae* clinical isolates while all the 82 non-*K. pneumoniae* clinical isolates were negative. Plasmids with the specific gene and the mouse blood with *K. pneumoniae* were used for sensitivity analysis. The detection limit of the LAMP was 1 bacterium/reaction. The results showed that the LAMP targeted to *ureR_1* is a fast, specific, sensitive, inexpensive, and suitable method for the detection of *K. pneumoniae*.

## Introduction

*Klebsiella pneumoniae* is a Gram-negative facultative anaerobic bacterium and an important conditional pathogen in hospitals over the past years (1–3). *K. pneumoniae* ubiquitously occurs in natural environments and is frequently found in the respiratory and gastrointestinal organs of patients (4,5). Moreover, it is one of the main causes of nosocomial infections, which can lead to pneumonia, urinary tract infection, and sepsis (6). Children, older adults, hypoimmune individuals, and the patients undergoing long-term antibiotic therapy and intensive treatment are the susceptible population to *K. pneumoniae*. The World Health Organization reported an estimated 18.8 billion cases of pneumonia, along with upper respiratory tract infections, and about 4 million deaths in 2013 (7).

The prompt delivery of definitive therapy can prevent the spread of *K. pneumoniae*, control the inflammatory process, and decrease the mortality rate (8). Hence, the efficient and rapid detection of *K. pneumoniae* is deemed important. Conventional methods for the identification of *K. pneumoniae* in clinics include bacterial culture, immunological methods, and polymerase chain reaction (PCR) (9–11). Bacterial culture was considered the gold standard procedure for identifying *K. pneumoniae* (12,13). After being infected by a pathogen, the specific antibody is formed in the host. Thus, immunological testing methods, including enzyme-linked immunosorbent assay, western blot, and immune chromatography, are based on the specificity of the antigen-antibody reaction. However, bacterial culture and immunological methods are time-consuming and exhibit low precision (14). Therefore, the PCR technique has been extensively used in clinical testing due to its high sensitivity and fast result (15). However, it is inconvenient in the clinical setting due to the requirement of isothermal cyclic amplification (16,17). Although whole genome sequencing has been recently identified as a precise method for studying pathogenic bacteria, the high costs of the necessary reagent and apparatus limit its universal application (18).

Loop-mediated isothermal amplification (LAMP) can be potentially used as a simple screening assay in the field by clinicians (1,19). Compared with the other methods, LAMP technology has the advantages of simplicity, rapidity, and high sensitivity. Thus, it is more suitable for use in clinical laboratories. A novel specific gene of *K. pneumoniae* was obtained through bioinformatics analysis in this study (*ureR_1*) and an efficient and accurate LAMP method to detect it was built for the detection of *K. pneumoniae,* and validated by PCR assay.

## Material and Methods

### Bacterial strains, culture conditions, and DNA extraction

In this study, 140 *K. pneumoniae* clinical isolates and 82 non-*K. pneumoniae* strains were obtained from the First People's Hospital of Yunnan Province. The 82 non-*K. pneumoniae* strains included *Escherichia coli* (n=15), *Staphylococcus aureus* (n=8), *Staphylococcus epidermidis* (n=24), *Enterococcus faecalis* (n=20), and *Micrococcus luteus* (n=15). All strains were collected by the doctors from the Hospital and cultured in Luria-Bertani medium in an orbital shaker (37°C, with 180 rpm, overnight). A bacterial genomic DNA kit (Zomanbio, China) and a direct-extraction reagent (20) were used to extract genomic DNA from bacteria. The extracted genomic DNA was stored at –20°C for further use.

### Screening of *K. pneumoniae* specific genes

First, the genomic sequence of *K. pneumoniae subsp. pneumoniae HS11286* (GenBank No. NC_016845.1) and the known non-redundant nucleic acid database from National Center for Biotechnology Information (NCBI) were downloaded to the local server as the local database. Then, the potential specific genes of *K. pneumoniae subsp. pneumoniae HS11286* were screened by sequence similarity alignment. Online BLAST was used to further identify the screened potential specific genes due to the slow update of the local database. Two-step strategies using interspecies-specific and intraspecies commonality were used to identify specific genes by online BLAST. The first one excluded *K. pneumoniae* during the alignment; the gene may be considered a possible target gene if the alignment result is different. The second included *K. pneumoniae* during alignment, and the highly conserved genes can be considered possible target genes. The retrieval range was limited to the species with known sequences except for *K. pneumoniae.* The specific gene of *K. pneumoniae* can only be considered when the alignment differs from those of other species, similar to those of a few species, or features a very low similarity.

### Primers design and reaction

Four oligonucleotide primers (outer and inner primers, F3/B3 and FIP/BIP, respectively) targeting the specific gene were designed by the Primer Explorer V5 software (http: //primerexplorer.jp/lampv5/index.html) for LAMP assay. The outer primers (B3/F3) were also used in the PCR assays; the target fragment of the amplification was 203 base pairs (bp). Table 1 presents the primers used in this study. PCR was performed using 2× TSINGKE Master Mix, which was purchased from TSINGKE Biological Technology Company (China). According to the operating instructions, the PCR reaction system containing 12.5 µL of 2× TSINGKE Master Mix, 1.0 µL of primers (10 µM), and 1.0 µg of DNA template was added with nuclease-free water up to 25 µL volume. The reactions were performed in a GeneAmp PCR System 9700 (Thermo Fisher Scientific, Inc., USA) with the following amplification conditions: pre-denaturation at 95°C for 5 min, followed by 32 cycles, denaturation at 95°C for 30 s, annealing at 57°C for 30 s, extension at 72°C for 30 s, and a final extension at 72°C for 7 min. Five microliters of the PCR products were used in the 2% agarose gel electrophoresis at 120 V, 30 min, and the agarose gel was stained by Gel stain (Beijing Transgen Biotech Co., Ltd., China). The LAMP reaction was carried out in a primer with a total volume of 25 µL. The system contained 12.5 μL 2× Isothermal Master Mix (Great Britain), 8 μM FIP and BIP, 1 μM B3 and F3, 100 ng genomic DNA, and up to 25 μL nuclease-free water. The reaction was amplified using Genie^®^ II (OptiGene, UK) at 65°C for 30 min, and then the primer was annealed at temperatures ranging from 80 to 89°C. SYBR-Green I (Beijing Solarbio Science & Technology Co., Ltd., China), a nuclear dye, was added into the LAMP reaction tubes for fluorescence visualization of the LAMP products.

**Table 1:** Primers for the amplification of the *ureR_1* gene.

Primers	Sequence (5′- 3′)	Size (bp)
Outer primers		
F3	CCGATAGAGAACTCGAACTG	20
B3	TCTGATGCATTTTACCCTGAT	21
Inner primers		
FIP	TCTTTGAAAAACCTTCGCTCCATATTTTTCTTCGCGCTAACTATCAACT	49
BIP	CATTCATATTGAAAAGCAGACCCGTTTTGCTCGATAAAGCCATGAGAA	48

### Construction of plasmids and preparation of blood template

The positive plasmids were constructed by the following steps. The DNA fragment of *ureR_1* was obtained by PCR, and the genome DNA of *K. pneumoniae* was used as a template. The *ureR_*1 gene was then inserted into the pMD 19-T simple vector and transformed into *JM109* competent cells. The positive clones were selected from the LB solid medium after overnight culture and used for plasmid extraction. Finally, the copies of recombinant plasmids were calculated by the deduced polynomial model described by the equation

$$C=\frac{X\times 10^{-9}}{A+Y\times 3 2 4}\times N_{A}$$

where C denotes the copies of plasmids; *X* and *Y* represent the concentration of plasmids and the number of base pairs of the target fragment, respectively; N_A_ is Avogadro's constant (6.02×10^23^); *A* is the number of base pairs of the vector; and 324 represents the average molecular weight of each base pair.

To prepare the simulated samples from patients, *K. pneumoniae* suspension was mixed with whole blood from mice at a volume ratio of 1:1. The mixed blood sample was then lysed using a direct-extraction reagent for PCR and LAMP assays.

### Specificity of the PCR and LAMP reactions

A total of 140 *K. pneumoniae* clinical isolates and 82 non-*K. pneumoniae* strains were used for the assessment of the PCR and LAMP reactions specificity. All genomic DNA of the tested strains were prepared by the TIANamp genomic DNA kit (Tiangen, China) for PCR and LAMP assays. The PCR test was used as the gold standard in preliminary experiments for the specificity test prior to the LAMP test. The LAMP test was used after validating the specificity by PCR.

### Sensitivity of the PCR and LAMP reactions

In this study, the sensitivity of PCR and LAMP reactions were evaluated using two different templates, the serially diluted 10-fold positive plasmids (10^9^−10^0^ copies) and the blood sample (10^7^−10^0^ bacteria) mimicking infection. The counted *K. pneumoniae* strain was serially diluted by 10-fold and then mixed with mouse blood at 1:1 proportion to mimic infection. A heating method was used to extract DNA from the blood sample and the suspension was used as the template for PCR and LAMP assays.

## Results

### Screening the specific gene

Following the preliminary screening of possible specific genes by local BLAST, we obtained 700 potential specific genes. These genes were then used in the second screening by online BLAST. Conclusively, 4 potential specific genes were attained, and they met the criterion about the interspecific specificity and intraspecies universality. The primers for LAMP reaction were designed on the Primer Explorer V5 software (http: //primerexplorer.jp/lampv5/index.html). In this step, four primers were designed for the six regions of the target gene in the LAMP, and the primers had to be confirmed by primer BLAST and PCR assay. With the above harsh selection conditions, the *ureR_1* gene (GenBank ID: 11847803) was finally identified as the only one that can be used in the detection of *K. pneumoniae*. The *ureR_1* gene was involved in the encoding of putative helix-turn-helix AraC-type transcriptional regulator (YP_005227085), and could be a new target for the identification of *K. pneumoniae*.

### Construction of the positive plasmid

Positive plasmids with *ureR_1* gene were constructed and verified by bacterial liquid PCR and sequencing. The positive clones were then cultured again for the extraction of positive plasmids. The plasmids were verified by agarose gel electrophoresis and stored at –20°C for future use.

### Specificity of the PCR and LAMP reactions

**Figure 1.**
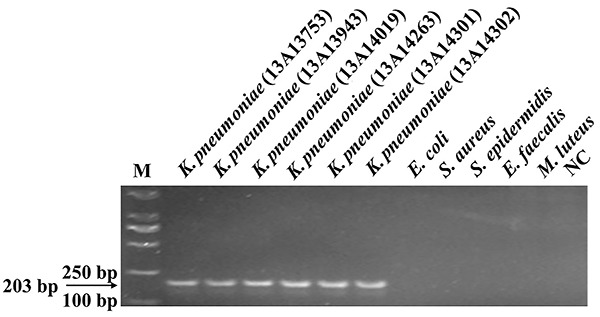
Specificity of the PCR assay for detecting the target gene *ureR_1* using the primers B3/F3. Genomic DNA of *K. pneumoniae* was used as the template for PCR in lane 2 to lane 7; the template in lane 8 to lane 12 were ordinal of control strains, *E. coli*, *S. aureus*, *S. epidermidis*, *E. faecalis*, *M. luteus*. M: 2000 marker; NC: negative control, with sterile distilled water as the template. All experiments were repeated twice.

**Figure 2.**
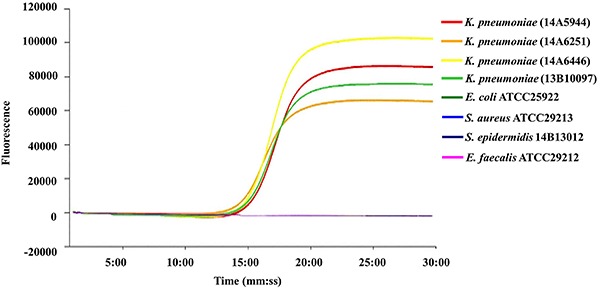
The amplification curve results of loop-mediated isothermal amplification (LAMP) specificity reaction. Specificity of the LAMP assay for detecting the target gene of *ureR_1* by Genie^®^ II. Genomic DNA of *K. pneumoniae* was used as the template for the LAMP test. All experiments were repeated twice.

**Figure 3.**
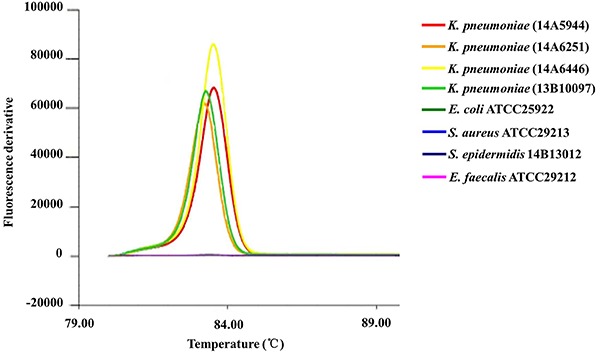
The melt curve in loop-mediated isothermal amplification (LAMP) specificity reaction tubes. Specificity of the LAMP assay for detecting the target gene of *ureR_1* by Genie^®^ II. Genomic DNA of *K. pneumoniae* was used as the template for LAMP test. All experiments were repeated twice.

**Figure 4.**
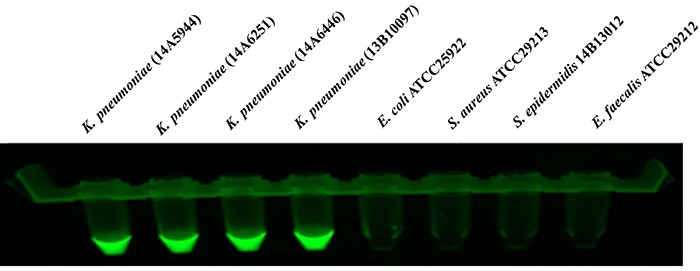
The fluorescence visualization of the loop-mediated isothermal amplification (LAMP) specificity reaction tubes. Specificity of the LAMP assay for detecting the target gene of *ureR_1*. Genomic DNA was used as the template for the LAMP. The results were observed under UV-light. All experiments were repeated twice.

Prior to LAMP reaction, the PCR test was used for the specificity test. One hundred and forty *K. pneumoniae* strains and 82 non-*K. pneumoniae* strains were tested. All the *non-K. pneumoniae* were negative and all the *K. pneumoniae* strains were positive, the representative results are shown in Figure 1. After validation by PCR, LAMP was used to test the specificity. Figure 2 shows the amplification curve results of the LAMP, and Figure 3 shows the melt curve of the products in LAMP specificity reaction tubes. Figure 4 shows the fluorescence visualization of the LAMP reaction tubes, which were consistent with PCR results.

### Sensitivity of the PCR and LAMP reactions

**Figure 5.**
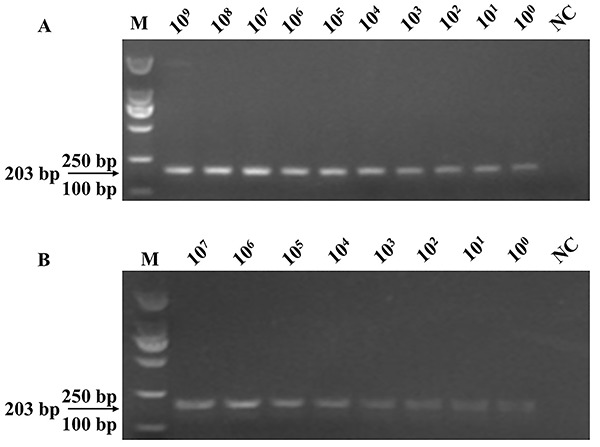
**A**, Sensitivity of the PCR assay for detecting the target gene of *ureR_1* using the primers FIP/BIP. The positive plasmids of *K. pneumoniae* were serially diluted 10-fold as templates for PCR assay. **B**, Bacterial solutions were serially diluted 10-fold with the mouse blood with a volume ratio of 1:1. These mixtures were lysed and then the suspension was used for PCR assay. M: 2000 marker; NC: negative control. All experiments were repeated twice.

**Figure 6.**
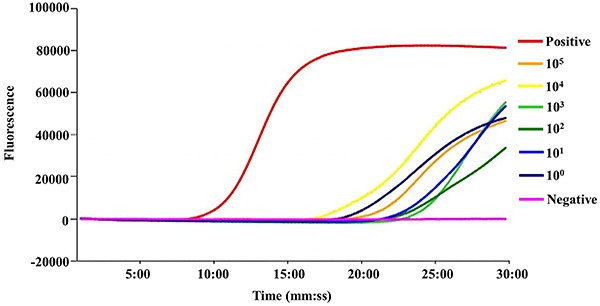
Sensitivity of the loop-mediated isothermal amplification (LAMP) reactions. The bacterial solutions were serially diluted 10-fold with the mouse blood with a volume ratio of 1:1. These mixtures were lysed and then the suspension was used for the LAMP assay. The positive plasmid was the positive control and water was the negative control. The concentration of bacterium was from 10^5^−10^0^. All the experiments were repeated twice.

**Figure 7.**
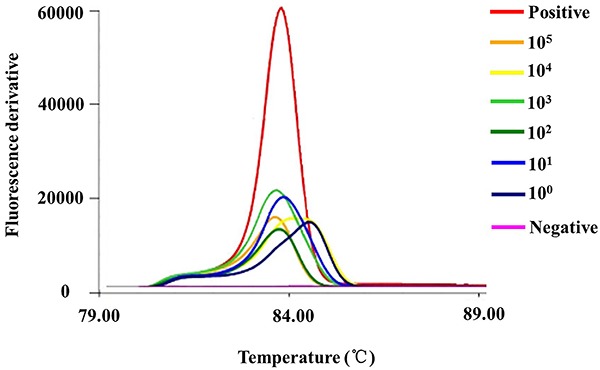
The melt curve of the products in loop-mediated isothermal amplification (LAMP) sensitivity reaction tubes for detecting the target gene of *ureR_1* by Genie^®^ II. The lysed suspension of *K. pneumoniae* and mouse blood were used as the template for LAMP test. The positive plasmid was the positive control and water was the negative control. The concentration of bacterium was from 10^5^−10^0^. All experiments were repeated twice.

**Figure 8.**
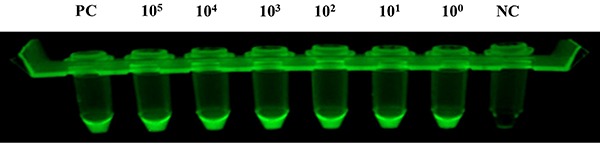
Fluorescence visualization of the loop-mediated isothermal amplification (LAMP) reaction tubes in the sensitivity test for detecting the target gene of *ureR_1*. The lysed suspension of *K. pneumoniae* and mouse blood was used as the template for the LAMP test. The positive plasmid was the positive control (PC) and water was the negative control (NC). The concentration of bacterium was from 10^5^−10^0^. The results were observed under UV-light. All experiments were repeated twice.

Figure 5 demonstrates the extreme sensitivities in PCR assay, which can reach up to 10^0^ copies per reaction. The LAMP results are shown in Figure 6. Figure 7 shows the melt curve of the products in LAMP sensitivity reaction tubes and Figure 8 shows the fluorescence visualization of the LAMP reaction tubes in the sensitivity test. According to Figures 5, 6, 7, and 8, both the PCR and LAMP assay exhibited a high sensitivity that can reach up to 10^0^ bacterium/reaction.

## Discussion

*K. pneumoniae* is one of the main pathogenic bacteria that causes acute respiratory infections, which is the primary cause of child mortality in developing countries, accounting for approximately 3 million deaths annually (7). In China, *K. pneumoniae* was reported to account for 9.03% of total bacterial infection in hospitals (21–23). In the United States, Europe, and Africa, *K. pneumoniae* has become the main pathogenic bacterium (after *Escherichia coli*) in patients with pyogenic liver abscess over the past two decades. *K. pneumoniae* infection exhibits the tendency to spread worldwide and poses a serious threat to the lives of people (22,24–26). Thus, early and accurate diagnosis can decrease the morbidity and mortality caused by *K. pneumoniae* infection, and a rapid and sensitive diagnostic method is urgently required.

Bacterial culture was the preferred method for identifying pathogenic bacterial infections in clinics. However, it was toilsome, slow, and poorly effective. Mass spectrometry is a high-speed approach used to detect pathogenic bacteria with higher accuracy than bacterial culture. However, mass spectrometry is expensive, and thus, it is not suitable for all types of hospitals. Numerous methods have been used to detect *K. pneumoniae* in clinics. PCR-based methods are widely used to detect *K. pneumoniae* by amplifying specific genes, including *rcsA*, *tyrB*, *gapA*, and 16S rRNA ITS (27–30). However, a high-precision thermal cycler is needed, which cannot be afforded by low-level medical institutions. By contrast, LAMP, which exhibits fast and accurate characteristics, has been widely used in clinical laboratories to detect pathogens, including bacteria, viruses, fungi, and parasites (31–33), although this technology is relatively new.

In the present work, a conservative specific gene was identified to detect *K. pneumoniae*. The results in Figure 1 suggest that the *ureR_1* gene was specific to *K. pneumoniae* and can be used as a biomarker in clinics. In many studies, the sensitivity of LAMP was reported to be 10-fold higher than that of the PCR method (27,29,34). However, specificity and sensitivity between LAMP and PCR were similar when the *ureR_1* gene was used. These results suggest that the *ureR_1* gene and the primers designed in this study may be more specific and sensitive than those in other studies (27,35). Also, only 32 min was needed for the identification of *K. pneumoniae* by the LAMP method, while 90 min are required in PCR. In addition, the LAMP reaction results can be easily observed under UV-light. The LAMP method can greatly reduce the time required for identification.

In conclusion, we identified *ureR_1* as a specific gene of *K. pneumoniae* and established a rapid, specific, and sensitive LAMP method using *ureR_1* primers for the detection of *K. pneumoniae.* The established method may be extensively used in clinics in the future due to its high specificity and sensitivity, easy visualization, and rapid results.

## References

[1] Lai YC, Peng HL, Chang HY (2003). RmpA2, an activator of capsule biosynthesis in Klebsiella
pneumoniae CG43, regulates K2 cps gene expression at the transcriptional
level. J Bacteriol.

[B02] Yang XJ, Wang S, Cao JM, Hou JH (2016). Draft genome sequence of klebsiella pneumoniae strain as isolated
from Zhejiang Provincial Hospital of TCM, China. Genome Announc.

[3] Kumar V, Park S (2018). Potential and limitations of Klebsiella pneumoniae as a microbial
cell factory utilizing glycerol as the carbon source. Biotechnol Adv.

[4] Podschun R, Ullmann U (1998). Klebsiella spp. as nosocomial pathogens: epidemiology, taxonomy,
typing methods, and pathogenicity factors. Clin Microbiol Rev.

[5] Bednarz-Misa I, Serek P, Dudek B, Pawlak A, Bugla-Ploskonska G, Gamian A (2014). Application of zwitterionic detergent to the solubilization of
Klebsiella pneumoniae outer membrane proteins for two-dimensional gel
electrophoresis. J Microbiol Methods.

[6] Rock C, Thom KA, Masnick M, Johnson JK, Harris AD, Morgan DJ (2014). Frequency of Klebsiella pneumoniae carbapenemase (KPC)-producing
and non-KPC-producing Klebsiella species contamination of healthcare workers
and the environment. Infect Control Hosp Epidemiol.

[7] Ferkol T, Schraufnagel D (2014). The global burden of respiratory disease. Ann Am Thoracic Soc.

[8] Wang F, Li R, Shang Y, Wang C, Wang GQ, Zhou DX (2016). A pilot study of quantitative loop-mediated isothermal
amplification-guided target therapies for hospital-acquired
pneumonia. Chin Med J.

[9] Bachman MA, Breen P, Deornellas V, Mu Q, Zhao L, Wu W (2015). Genome-wide identification of Klebsiella pneumoniae fitness genes
during lung infection. mBio.

[B10] Pongsachareonnont P, Honglertnapakul W, Chatsuwan T (2017). Comparison of methods for identifying causative bacterial
microorganisms in presumed acute endophthalmitis: conventional culture,
blood culture, and PCR. BMC Infect Dis.

[11] Ahn JG, Choi SY, Kim DS, Kim KH (2012). Enhanced detection and serotyping of Streptococcus pneumoniae
using multiplex polymerase chain reaction. Korean J Pediatr.

[12] Hudu SA, Alshrari AS, Syahida A, Sekawi Z (2016). Cell culture, technology: enhancing the culture of diagnosing
human diseases. J Clin Diagn Res.

[13] Kloss S, Kampe B, Sachse S, Rosch P, Straube E, Pfister W (2013). Culture independent Raman spectroscopic identification of urinary
tract infection pathogens: a proof of principle study. Anal Chem.

[14] Nakano R, Nakano A, Ishii Y, Ubagai T, Kikuchi-Ueda T, Kikuchi H (2015). Rapid detection of the Klebsiella pneumoniae carbapenemase (KPC)
gene by loop-mediated isothermal amplification (LAMP). J Infect Chemother.

[15] Abdoli A, Dalimi A, Soltanghoraee H, Ghaffarifar F (2016). Molecular detection of Toxoplasma gondii in house sparrow
(*Passer domesticus*) by LAMP and PCR methods in Tehran,
Iran. J Parasit Dis.

[16] Ali ME, Razzak MA, Hamid SB, Rahman MM, Amin MA, Rashid NR (2015). Multiplex PCR assay for the detection of five meat species
forbidden in Islamic foods. Food Chem.

[17] Bharathi MJ, Murugan N, Rameshkumar G, Ramakrishnan R, Venugopal Reddy YC, Shivkumar C (2013). Comparative evaluation of uniplex, nested, semi-nested, multiplex
and nested multiplex PCR methods in the identification of microbial etiology
of clinically suspected infectious endophthalmitis. Curr Eye Res.

[18] Long SW, Williams D, Valson C, Cantu CC, Cernoch P, Musser JM (2013). A genomic day in the life of a clinical microbiology
laboratory. J Clin Microbiol.

[19] Notomi T, Okayama H, Masubuchi H, Yonekawa T, Watanabe K, Amino N (2000). Loop-mediated isothermal amplification of DNA. Nucleic Acids Res.

[20] Brewster JD, Paoli GC (2013). DNA extraction protocol for rapid PCR detection of pathogenic
bacteria. Anal Biochem.

[21] Miriagou V, Cornaglia G, Edelstein M, Galani I, Giske CG, Gniadkowski M (2010). Acquired carbapenemases in Gram-negative bacterial pathogens:
detection and surveillance issues. Clin Microbiol Infect.

[22] Liu Y, Wang JY, Jiang W (2013). An increasing prominent disease of klebsiella pneumoniae liver
abscess: etiology, diagnosis, and treatment. Gastroenterol Res Pract.

[23] Basu S (2009). Klebsiella pneumoniae: An emerging pathogen of pyogenic liver
abscess. Oman Med J.

[24] Yonetani S, Ohnishi H, Ohkusu K, Matsumoto T, Watanabe T (2016). Direct identification of microorganisms from positive blood
cultures by MALDI-TOF MS using an in-house saponin method. Int J Infect Dis.

[B25] Hou Y, Zhang X, Hou X, Wu R, Wang Y, He X (2018). Rapid pathogen identification using a novel microarray-based
assay with purulent meningitis in cerebrospinal fluid. Sci Rep.

[26] Alfaresi M, Mahboub B (2017). Identification of bacteria in the sputum of a cystic fibrosis
patient; a comparison of phenotypic and molecular methods. Open Microbiol J.

[27] Dong D, Liu W, Li H, Wang Y, Li X, Zou D (2015). Survey and rapid detection of Klebsiella pneumoniae in clinical
samples targeting the rcsA gene in Beijing, China. Front Microbiol.

[B28] Hirama T, Minezaki S, Yamaguchi T, Kishi E, Kodama K, Egashira H (2014). HIRA-TAN: a real-time PCR-based system for the rapid
identification of causative agents in pneumonia. Respir Med.

[29] Jeong ES, Lee KS, Heo SH, Seo JH, Choi YK (2013). Rapid identification of Klebsiella pneumoniae, Corynebacterium
kutscheri, and Streptococcus pneumoniae using triplex polymerase chain
reaction in rodents. Exp Anim.

[30] Tocchioni F, Tani C, Bartolini L, Moriondo M, Nieddu F, Pecile P (2016). The role of dna amplification and cultural growth in complicated
acute appendicitis. Pediatr Rep.

[31] Ohtsuka K, Yanagawa K, Takatori K, Hara-Kudo Y (2005). Detection of Salmonella enterica in naturally contaminated liquid
eggs by loop-mediated isothermal amplification, and characterization of
Salmonella isolates. Appl Environ Microbiol.

[B32] Ptaszynska AA, Borsuk G, Wozniakowski G, Gnat S, Malek W (2014). Loop-mediated isothermal amplification (LAMP) assays for rapid
detection and differentiation of Nosema apis and N. ceranae in
honeybees. FEMS Microbiol Lett.

[33] Soliman H, Saleh M, El-Matbouli M (2015). Detection of fish pathogens by loop-mediated isothermal
amplification (LAMP) technique. Methods Mol Biol.

[34] Kitamura M, Aragane M, Nakamura K, Watanabe K, Sasaki Y (2017). Rapid identification of drug-type strains in Cannabis sativa
using loop-mediated isothermal amplification assay. J Nat Med.

[35] Kinoshita Y, Niwa H, Katayama Y (2015). Use of loop-mediated isothermal amplification to detect six
groups of pathogens causing secondary lower respiratory bacterial infections
in horses. Microbiol Immunol.

